# Automated Tongue Feature Extraction for ZHENG Classification in Traditional Chinese Medicine

**DOI:** 10.1155/2012/912852

**Published:** 2012-05-31

**Authors:** Ratchadaporn Kanawong, Tayo Obafemi-Ajayi, Tao Ma, Dong Xu, Shao Li, Ye Duan

**Affiliations:** ^1^Department of Computer Science and Informatics Institute, University of Missouri, Columbia, MO 65211, USA; ^2^MOE Key Laboratory of Bioinformatics and Bioinformatics Division, TNLIST/Department of Automation, Tsinghua University, Beijing 100084, China

## Abstract

ZHENG, Traditional Chinese Medicine syndrome, is an integral and essential part of Traditional Chinese Medicine theory. It defines the theoretical abstraction of the symptom profiles of individual patients and thus, used as a guideline in disease classification in Chinese medicine. For example, patients suffering from gastritis may be classified as Cold or Hot ZHENG, whereas patients with different diseases may be classified under the same ZHENG. Tongue appearance is a valuable diagnostic tool for determining ZHENG in patients. In this paper, we explore new modalities for the clinical characterization of ZHENG using various supervised machine learning algorithms. We propose a novel-color-space-based feature set, which can be extracted from tongue images of clinical patients to build an automated ZHENG classification system. Given that Chinese medical practitioners usually observe the tongue color and coating to determine a ZHENG type and to diagnose different stomach disorders including gastritis, we propose using machine-learning techniques to establish the relationship between the tongue image features and ZHENG by learning through examples. The experimental results obtained over a set of 263 gastritis patients, most of whom suffering Cold Zheng or Hot ZHENG, and a control group of 48 healthy volunteers demonstrate an excellent performance of our proposed system.

## 1. Introduction

Traditional Chinese Medicine (TCM) has a long history in the treatment of various diseases in East Asian countries and is also a complementary and alternative medical system in Western countries. TCM takes a holistic approach to medicine with emphasis on the integrity of the human body and the close relationship between a human and its social and natural environment [1]. TCM applies different therapeutic methods to enhance the body's resistance to diseases and prevention. TCM diagnosis is based on the information obtained from four diagnostic processes, that is, *looking, listening, and smelling, asking, and touching.* The most common tasks are taking the pulse and inspecting the tongue [2]. For thousands of years, Chinese medical practitioners have diagnosed the health status of a patients' internal organs by inspecting the tongue, especially the patterns on the tongue's surface. The tongue mirrors the viscera. The changes of tongue can objectively manifest the states of a disease, which can help differentiate syndromes, establish treatment methods, prescribe herbs, and determine prognosis of disease.

ZHENG (TCM syndrome) is an integral and essential part of TCM theory. It is a characteristic profile of all clinical manifestations that can be identified by a TCM practitioner. ZHENG is an outcome after analyzing all symptoms and signs (tongue appearance and pulse feeling included). All diagnostic and therapeutic methods in TCM are based on the differentiation of ZHENG, and this concept is as ancient as TCM in China [3]. ZHENG is not simply an assemblage of disease symptoms but rather can be viewed as the TCM theoretical abstraction of the symptom profiles of individual patients. *As noted in the abstract, ZHENG is also used as a guideline in TCM disease classification. For example, patients suffering from the same disease may be grouped into different ZHENGs, whereas different diseases may be grouped as the same ZHENG. The Cold ZHENG (Cold syndrome) and the Hot ZHENG (Cot syndrome) are the two key statuses of ZHENG *[3].* Other ZHENGs include Shen-Yang-Xu ZHENG (Kidney-Yang deficiency syndrome), Shen-Xu ZHENG (Kidney deficiency syndrome), and Xue-Yu ZHENG (Blood Stasis syndrome)* [4].

In this paper, we explore new modalities for the clinical characterization of ZHENG using various supervised machine-learning algorithms. Using an automated tongue-image diagnosis system, we extract objective features from tongue images of clinical patients and analyze the relationship with their corresponding ZHENG data and disease prognosis (specifically stomach disorders, i.e., gastritis) obtained from clinical practitioners. We propose a system that learns from the clinical practitioner's subjective data on how to classify a patient's health status by extracting meaningful features from tongue images using a rich set of features based on color-space models. Our premise is that Chinese medical practitioners usually observe the tongue color and coating to determine ZHENG such as Hot or Cold ZHENG, and to diagnose different stomach disorders including gastritis. Hence, we propose using machine-learning techniques to establish the relationship between the tongue image features and the ZHENG by learning through examples. We are also interested in the correlation between the Hot and Cold patterns observed in ZHENG gastritis patients and their corresponding symptom profiles.

Various types of features have been explored for tongue feature extraction and tongue analysis, including texture [5], color [6–8], shape [9], spectrum [8], among others. A systematic tongue feature *set, comprising of a combination of geometric features (size, shape, etc.), cracks, and textures, was later proposed by Zhang et al.* [10]. Computer-aided tongue analysis systems based on these types of features have also been developed [11, 12]. Our goal is to provide a set of objective features that can be extracted from patients' tongue images, based on the knowledge of ZHENG, which improves accuracy of an objective clinical diagnosis. Our proposed tongue feature set is based on an extensive color model.

This paper is organized as follows: in Section 2, we provide a TCM descriptive view of the physiology of the tongue. An overview of the proposed feature extraction and learning framework along with a complete description of the color space model feature set is presented in Section 3. Our experimental results and analysis in a tongue image dataset from gastritis patients with Cold ZHENG and Hot ZHENG are discussed in Section 4 before drawing our conclusions and proposing plans for future work in Section 5.

## 2. Tongue Diagnosis in TCM

**Figure 1:**
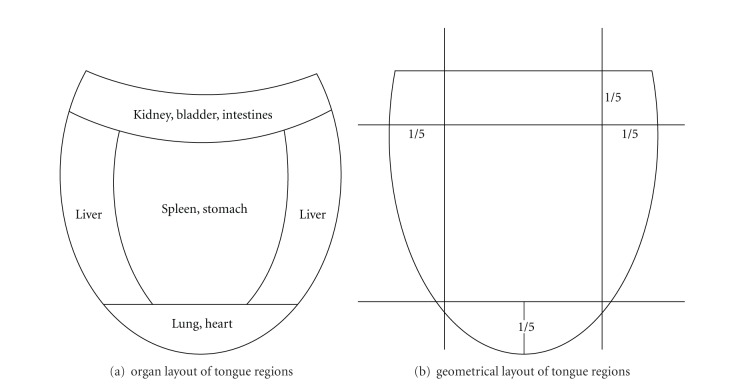
Tongue areas and their correspondence to internal organs in TCM.

TCM believes that the tongue has many relationships and connections in the human body, both to the meridians and the internal organs. It is, therefore, very useful and important during inspection for confirming TCM diagnosis as it can present strong visual indicators of a person's overall physical and mental harmony or disharmony. In TCM, the tongue is divided into tongue tip, tongue margins, tongue center, and tongue root. *Figure 1(a) shows each part of the tongue and its correspondence to different internal organs according to TCM while Figure 1(b) illustrates how we geometrically obtain an approximation of these regions from the tongue image.* The tongue tip reflects the pathological changes in the heart and lungs, while the bilateral sides of the tongue reflect that of the liver and gallbladders. The pathological changes in the spleen and stomach are mirrored by the center of tongue, while changes in the kidneys, intestines, and bladder section correspond to the tongue root.

*In this paper, we focus on the patients with stomach disorders, gastritis. Hence, we are interested in extracting features not only from entire tongue image but also specifically from the middle region, as this corresponds to the stomach organ, according to TCM. We extract the middle rectangular region, illustrated in Figure 1(b), as our approximation for the tongue middle region.*

The practitioner examines the general and local shape as well as the color of the tongue and its coating. According to TCM, the normal tongue is pale red with thin white coating. Some signs of imbalance or pathology are red body, yellow coating, or thick coating like mozzarella cheese, and so forth. Some characteristic changes occur in the tongue in some particular diseases. Most tongue attributes are on the tongue surface. A TCM doctor looks at several attributes of tongue body: color, moisture, size, shape, and coating. These signs not only reveal overall states of health but they also correlate to specific organ functions and disharmonies, especially in the digestive system.

The two main characteristics of the tongue in TCM ZHENG diagnosis are the color and the coating. The color of the patient's tongue color provides information about his/her health status. For example [13], dark red color can indicate inflammation or ulceration, while a white tongue indicates cold attack, mucus deposits, or a weakness in the blood leading to such conditions as anemia [12]. Moreover, a yellow tongue points out a disorder of the liver and gallbladder, and blue or purple implies stagnation of blood circulation and a serious weakening of the part of the digestive system that corresponds to the area of the tongue where the color appears.

**Figure 2:**
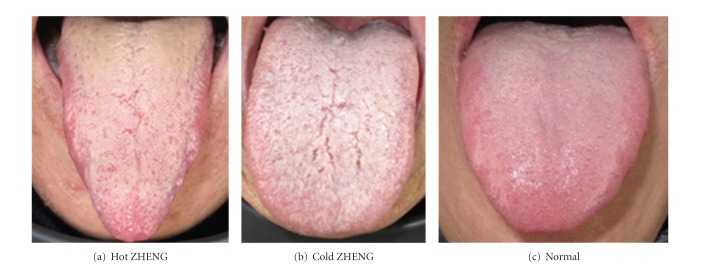
Tongue images of patients with different ZHENG classification. “Normal” represents a healthy person.

The coating on the tongue is discriminated by not only its presence but also its color. The color could be yellow, white, and other colors. However, the color in image is not the exact true color of the tongue. To properly identify the color of the tongue coating, we applied the specular component technique presented in our prior work on tongue detection and analysis [2].  Figure 2 illustrates different tongue images of patients and their corresponding ZHENG class.

## 3. Tongue Feature Extraction and Classification Framework

### 3.1. Feature Extraction for Tongue Image Analysis

Our goal is to compute a set of objective features ${\vec{F}}_{j}=\left\{F_{n}\right\}$ from each tongue image *j *that can be fed into our learning system so that we can predict not only the color and coating on the tongue, but also different ZHENGs of the gastritis patients. These features are designed to capture different color characteristics of the tongue. While a single feature may not be very discriminative, our premise is that the aggregation of these features will be discriminative. We leave it to the learning algorithm to determine the weight/contribution of each feature in the final classification.

Most color spaces are represented in tuples of number, normally three or four color components. Color components determine the position of the color in the color space used. There are many color spaces defined for different purposes. We designed a set of 25 features that span the entire color-space model. They can be grouped under eight categories: RGB, HSV, YIQ, Y'CbCr, XYZ, L*a*b*, CIE Luv, and CMYK.

In this section, we first describe in detail how we compute each feature ${\vec{f}}^{i}$ per *i*th pixel in the image. Then, we explain how each feature per pixel is aggregated to obtain ${\vec{F}}_{j}=\{F_{n}\}$ per tongue image *j.*

#### 3.1.1. RGB

RGB is an additive color system, based on trichromatic theory in which red, green, and blue light components are added together to produce a specific pigment. The RGB model encodes the intensity of red, green, and blue, respectively. (*R*_*i*_, *G*_*i*_, *B*_*i*_) for each pixel is an unsigned integer between 0 and 255. Each RGB feature {*f*_*n*_^*i*^ | *n* = 1,…, 3} represents the normalized intensity value of the red, green, and blue component, respectively, of the  *i*th pixel in the image. We denote the normalized value of each component as  *r*_*i*_ = *R*_*i*_/255, *g*_*i*_ = *G*_*i*_/255, and  *b*_*i*_ = *B*_*i*_/255. Thus, *f*_1_^*i*^ = *r*_*i*_; *f*_2_^*i*^ = *g*_*i*_; *f*_3_^*i*^ = *b*_*i*_.

All the remaining color-space model features described in our feature set derive their value from the RGB feature set.

#### 3.1.2. HSV

HSV color space represents color using a 3-tuple set of hue, saturation, and value. It separates the luminance component of the color from chrominance information. The HSV model (*H*_*i*_, *S*_*i*_, *V*_*i*_) is obtained by a linear transformation of thenormalized RGB color space {*r*_*i*_, *g*_*i*_, *b*_*i*_}.

For each pixel *p*_*i*_, let ${\tilde{M}}_{i}=\max\left\{r_{i},g_{i},b_{i}\right\}$ represent the maximum value of the pixel's RGB triple set while ${\tilde{m}}_{i}=\min(r_{i},g_{i},b_{i})$, the minimum value of the set. We also denote the difference between maximum and minimum values of each RGB tuple by $\Delta_{i}\;={\tilde{M}}_{i}-{\tilde{m}}_{i}$. The HSV components {*H*_*i*_, *S*_*i*_, *V*_*i*_} are computed from RGB color space {*r*_*i*_, *g*_*i*_, *b*_*i*_} as follows:

(1) $$\begin{array}{c} V_{i}=\tilde{M_{i}},\\ S_{i}=\{\begin{array}{cc} 0, & {\tilde{M}}_{i}=0,\\ \frac{\Delta_{i}}{{\tilde{M}}_{i}}, & \text{otherwise}, \end{array}\\ H_{i}=\{\begin{array}{cc} 0, & \Delta_{i}=0,\\ \frac{g_{i}-b_{i}}{6\cdot \Delta_{i}}, & {\tilde{M}}_{i}=r_{i},\\ \left(\frac{b_{i}-r_{i}}{\Delta_{i}}+2\right)\cdot \frac{1}{6}, & {\tilde{M}}_{i}=g_{i},\\ \left(\frac{r_{i}-g_{i}}{\Delta_{i}}+4\right)\cdot \frac{1}{6}, & {\tilde{M}}_{i}=b_{i}. \end{array} \end{array}$$

Thus, the HSV features are *f*_4_^*i*^ = *H*_*i*_; *f*_5_^*i*^ = *S*_*i*_; *f*_6_^*i*^ = *V*_*i*_.

#### 3.1.3. YIQ

The YIQ color model is the television transmission color space for a digital standard. The *Y* component represents the perceived luminance, while *I* and *Q* components are the color information. *I* character is referred to “in-phase” term and *Q* letter stands for “quadrature.” *I* and *Q* can place color in a graph representing *I* as *X* axis and *Q* as *Y* axis. The YIQ system takes advantage of human color perceiver characteristics [14, 15]. 

The YIQ model (*Y*_*i*_, *I*_*i*_, *Q*_*i*_) is obtained by a linear transformation of the normalized RGB color space {*r*_*i*_, *g*_*i*_, *b*_*i*_} as follows:

(2) $$\left[\begin{array}{c} Y_{i}\\ I_{i}\\ Q_{i} \end{array}\right]=\left[\begin{array}{ccc} 0.299 & +0.587 & +0.114\\ 0.596 & -0.274 & -0.322\\ 0.211 & -0.523 & +0.312 \end{array}\right]\left[\begin{array}{c} r_{i}\\ g_{i}\\ b_{i} \end{array}\right].$$

The {*Y*_*i*_, *I*_*i*_, *Q*_*i*_} values are each normalized to obtain {*y*_*i*_, *i*_*i*_, *q*_*i*_} ∈ [0,1]. Thus, the YIQ features are *f*_7_^*i*^ = *y*_*i*_; *f*_8_^*i*^ = *i*_*i*_; *f*_9_^*i*^ = *q*_*i*_.

#### 3.1.4. Y'CbCr

Like YIQ, Y'CbCr is the television transmission color spaces but it is in analogue spaces for the NTSC system. YCbCr color space detaches RGB into the luma component, the blue-difference and red-difference chroma components. The transformation equation from RGB (unnormalized) model to YCbCr is defined as

(3) $$\left[\begin{array}{c} Y_{i}^{\prime }\\ {\text{Cb}}_{i}\\ {\text{Cr}}_{i} \end{array}\right]=\left[\begin{array}{ccc} 0.299 & +0.587 & +0.114\\ -0.169 & -0.331 & +0.500\\ 0.500 & -0.419 & -0.081 \end{array}\right]\left[\begin{array}{c} R_{i}\\ G_{i}\\ B_{i} \end{array}\right].$$

Similar to the YIQ features, the {*Y*_*i*_′, *Cb*_*i*_, *Cr*_*i*_} values are each normalized to obtain{*y*_*i*_′, *cb*_*i*_, *cr*_*i*_} ∈ [0,1]. Thus the YIQ features are *f*_10_^*i*^ = *y*_*i*_′; *f*_11_^*i*^ = *cb*_*i*_; *f*_12_^*i*^ = *cr*_*i*_.

#### 3.1.5. XYZ

Brightness and chromaticity are two principal components of color that interact with human vision. XYZ are developed under CIE XYZ color space [16]. The XYZ values can be obtained by a linear transformation of the gamma corrected value of the RGB normalized color space {*r*_*i*_, *g*_*i*_, *b*_*i*_}.

The gamma-corrected function is defined as

(4) $$\begin{array}{c} \gamma \left(t\right)=\{\begin{array}{cc} \frac{t}{12.92}, & \text{if}\;t\leq 0.04045,\\ {\left(\frac{t+a}{1+a}\right)}^{2.4}, & \text{otherwise}, \end{array} \end{array}$$

where  *a* = 0.055. Thus, XYZ model consisting of {*X*_*i*_, *Y*_*i*_′′, *Z*_*i*_} components is given by

(5) $$\left[\begin{array}{c} X_{i}\\ Y_{i}^{\prime \prime }\\ Z_{i} \end{array}\right]=\left[\begin{array}{ccc} 0.4124 & 0.3576 & 0.1805\\ 0.2126 & 0.7152 & 0.0722\\ 0.0193 & 0.1192 & 0.9505 \end{array}\right]\left[\begin{array}{c} \gamma \left(r_{i}\right)\\ \gamma \left(g_{i}\right)\\ \gamma \left(b_{i}\right) \end{array}\right].$$

The {*X*_*i*_, *Y*_*i*_′′, *Z*_*i*_} values are each normalized to obtain  {*x*_*i*_, *y*_*i*_′′, *z*_*i*_} ∈ [0,1]. Thus, the XYZ features are defined as *f*_13_^*i*^ = *x*_*i*_; *f*_14_^*i*^ = *y*_*i*_′′; *f*_15_^*i*^ = *z*_*i*_.

#### 3.1.6. L*a*b*

CIE L*a*b* color space is a nonlinear transformation of the CIE XYZ color space [17]. CIE L*a*b* try to imitate the logarithmic response of the human eye. The L* component is designed to match closely with human perception of lightness. The other two components describe the chroma. 

The forward transformation of CIE XYZ color space to CIE L*a*b* is computed as follows:

(6) $$\begin{array}{c} L_{i}^{*}=116\varphi \left(\frac{Y_{i}^{\prime \prime }}{\delta_{2}}\right)-16,\\ A_{i}=500\left[\varphi \left(\frac{X_{i}}{\delta_{1}}\right)-\varphi \left(\frac{Y_{i}^{\prime \prime }}{\delta_{2}}\right)\right],\\ B_{i}=200\left[\varphi \left(\frac{Y_{i}^{\prime \prime }}{\delta_{2}}\right)-\varphi \left(\frac{Z_{i}}{\delta_{3}}\right)\right], \end{array}$$

where

(7) $$\begin{array}{c} \varphi \left(t\right)=\{\begin{array}{cc} t^{1/3}, & \text{if}\;t>{\left(\frac{6}{29}\right)}^{3},\\ \frac{1}{3}{\left(\frac{29}{6}\right)}^{2}t+\frac{4}{29}, & \text{otherwise}, \end{array} \end{array}$$

and {*δ*} denotes the D65 white point given by {0.950456,1.0,1.088754}.

The L*a*b* values {L*_i_, *A*_*i*_, *B*_*i*_} are normalized as  {*l*_i_*, *a*_*i*_, *b*_*i*_} ∈ [0,1]. Hence, the CIE L*a*b* color features are given by *f*_16_^*i*^ = *l*_i_*; *f*_17_^*i*^ = *a*_*i*_; *f*_18_^*i*^ = *b*_*i*_.

#### 3.1.7. CIE Luv

CIE Luv, or L*u*v*, is color-space-computed from the transformation of the CIE XYZ color space by International Commission on Illumination (CIE) in order to perceptual uniformity [17]. Similar to CIE L*a*b*, the D65 white point is referred by {*δ*}:

(8) $$\begin{array}{c} L_{i}^{\prime \prime }=\{\begin{array}{cc} {\left(\frac{29}{3}\right)}^{3}\left(\frac{Y_{i}^{\prime \prime }}{\delta_{2}}\right), & \text{if}\;\frac{Y_{i}^{\prime \prime }}{\delta_{2}}\leq {\left(\frac{6}{29}\right)}^{3},\\ 116{\left(\frac{Y_{i}^{\prime \prime }}{\delta_{2}}\right)}^{1/3}-16, & \text{otherwise}, \end{array}\\ U_{i}=13L_{i}^{\prime \prime }\left(\frac{4X_{i}}{X_{i}+15Y_{i}^{\prime \prime }+3Z_{i}}-k_{1}\right),\\ V_{i}=13L_{i}^{\prime \prime }\left(\frac{9Y_{i}^{\prime \prime }}{X_{i}+15Y_{i}^{\prime \prime }+3Z_{i}}-k_{2}\right), \end{array}$$

where *k*_1_ = 0.2009,   *k*_2_ = 0.4610, under the standard luminance C. The normalized {*L*_*i*_′′, *U*_*i*_, *V*_*i*_} values are denoted by {*l*_*i*_′′, *u*_*i*_, *v*_*i*_} ∈ [0,1]. Therefore, *f*_19_^*i*^ = *l*_*i*_′′; *f*_20_^*i*^ = *u*_*i*_; *f*_21_^*i*^ = *v*_*i*_.

#### 3.1.8. CMYK

The CMYK color space is a subtractive color system mainly used in the printing industry [16]. The components consist of cyan, magenta, yellow, and neutral black. It is a common way to translate RGB display on monitors to CMYK values for printing.

Let ${\tilde{M}}_{i}=\max\left\{r_{i},g_{i},b_{i}\right\}$ represent the maximum value of the pixel's RGB triple set. The CMYK color space, denoted by {*C*_*i*_, *M*_*i*_, *Y*_i_*, *K*_*i*_}, can be computed from the RGB model as follows:

(9) $$\begin{array}{c} K_{i}=1-\;{\tilde{M}}_{i},\\ C_{i}=\frac{{\tilde{M}}_{i}-r_{i}}{{\tilde{M}}_{i}},\\ M_{i}=\frac{{\tilde{M}}_{i}-g_{i}}{{\tilde{M}}_{i}},\\ Y_{i}^{*}=\frac{{\tilde{M}}_{i}-b_{i}}{{\tilde{M}}_{i}}, \end{array}$$

Thus, the CMYK features are computed as *f*_22_^*i*^ = *C*_*i*_; *f*_23_^*i*^ = *M*_*i*_; *f*_24_^*i*^ = *Y*_i_*; *f*_25_^*i*^ = *K*_*i*_.

#### 3.1.9. Aggregate Operators for the Feature Vectors

To train our classification model using this set of features, we need to combine the features per pixel into one composite feature vector ${\vec{F}}_{j}=\{F_{n}\}$ per tongue image (or region) *j*. We aggregate the pixel features using two different statistical averages (mean and median) and the standard deviation values. We derive five variations of feature vectors for our automated tongue ZHENG classification system using the following operators: mean, median $\left(\text{med}\;\vec{F}\right)$, standard deviation ($\sigma \vec{F}$), “mean plus standard deviation” ($\left\{\mu \vec{F},\;\sigma \vec{F}\right\}$), and “median plus standard deviation” ($\left\{\text{med}\;\vec{F},\;\sigma \vec{F}\right\}$).

Let N denote the number of pixels in a given tongue image (or region) *j*. The mean feature vector is denoted by$\;\mu \vec{F_{j}}=\{\mu F_{n}\}$, where *μF*_*n*_ is given by

(10) $$\begin{array}{c} \mu F_{n}=\frac{\sum_{i=1}^{N}f_{n}^{i}}{N},\;n=1,\ldots ,\;25. \end{array}$$

The median feature vector, denoted by $\text{med}\;\vec{F_{j}}=\{\text{med}\;F_{n}\}$, is computed as med *F*_*n*_ = mid{sort(*F*_set_)}, *n* = 1,…, 25. Standard deviation depicts the margin of difference between a given feature value and its average value among all the pixels in the given region. Thus, the standard deviation feature vector is denoted by $\sigma \vec{F_{j}}=\{\sigma F_{n}\}$, where *σF*_*n*_ is given by

(11) $$\begin{array}{c} \sigma F_{n}=\sqrt[{2}]{\frac{\sum_{i=1}^{N}\left(f_{n}^{i}-\mu F_{n}\right)}{N}},\;n=1,\ldots ,\;25. \end{array}$$

The “mean plus standard deviation,” denoted by $\left\{\mu \vec{F},\;\sigma \vec{F}\right\}$, is a concatenation of the mean feature vector and the standard deviation feature vector. Similarly, the “median plus standard deviation” feature vector, denoted by $\left\{\text{med}\;\vec{F},\;\sigma \vec{F}\right\}$, is a concatenation of the median feature vector and the standard deviation feature vector. Thus, the total number of features in both concatenated feature vectors is 50 each.

### 3.2. Supervised Learning Algorithms for ZHENG Classification

We apply three different supervised learning algorithms (AdaBoost, support vector machine, multilayer perceptron network) to build classification models for training and evaluating the proposed automated tongue based diagnosis system. Each model has its strength and weakness, which we describe briefly below. We empirically evaluate their performance over our dataset.

#### 3.2.1. AdaBoost

An ensemble of classifiers is a set of classifiers whose individual predictions are combined in some way (typically by voting) to classify new examples. Boosting is a type of ensemble classifier which generates a set of weak classifiers using instances drawn from an iteratively updated distribution of the data, where in each iteration the probability of incorrectly classified examples is increased and the probability of the correctly classified examples is decreased. The ensemble classifier is a weighted majority vote of the sequence of classifiers produced.

The AdaBoost algorithm [18] trains a weak or base-learning algorithm repeatedly in a series of round *t* = 1,…, *T*. Given a training set {*x*_*i*_,  *y*_*i*_}_*i*=1,…,*n*_, where *x*_*i*_ belongs to some domain *X* and *y*_*i*_ ∈ *Y* = {−1, +1} (the corresponding binary class labels), we denote the weight of *i*th example in round *t* by *D*_*t*_(*i*). Initially, all weights are set equally and so *D*_1_(*i*) = 1/*n*, for all  *i*. For each round *t*, a weak learner is trained using the current distribution *D*_*t*_. When we obtain a weak hypothesis *h*_*t*_ with error *ϵ*_*t*_ = Pr_*i*~*D*_*t*__[*h*_*t*_(*x*_*i*_) ≠ *y*_*i*_.], if *ϵ*_*t*_ > 1/2, we end training; otherwise, we set *α*_*t*_ = (1/2)ln⁡((1 − *ϵ*_*t*_)/*ϵ*_*t*_) and update *D*_*t*+1_ as

(12) $$\begin{array}{c} D_{t+1}\left(i\right)=\frac{D_{t}\left(i\right)}{Z_{t}}\times \{\begin{array}{cc} e^{-\alpha_{t}} & \text{if}\;h_{t}\left(x_{i}\right)=y_{i},\\ e^{\alpha_{t}} & \text{if}\;h_{t}\left(x_{i}\right)\neq y_{i}, \end{array} \end{array}$$

where *Z*_*t*_ is a normalization factor. 

The final hypothesis is given by *H *(*x*) = sign⁡(∑_*t*=1_^*T*^ *a*_*t*_ *h*_*t*_(*x*)).

#### 3.2.2. Support Vector Machine

The support vector machine (SVM) [19] is one of the best-known general purpose learning algorithms. The goal of the SVM is to produce a model which predicts target values of data instances in the testing set given a vector of feature attributes. It attempts to maximize the margin of separation between the support vectors of each class and minimize the error in case the data is nonlinearly separable. The SVM classifiers usually perform well in high-dimensional spaces, avoid overfitting, and have good generalization capabilities. 

For a given a training set {*x*_*i*_,*y*_*i*_}_*i*=1,…,*n*_, the SVM model for an instance *x*  can be written as [20]

(13) $$f\left(x\right)=\overset{n}{\underset{i=1}{\sum }}{y_{i}\alpha }_{i}k\left(x_{i},x\right)+b,$$

where *k* is the kernel function used (polynomial kernel in this work), *α*_*i*_ is the Lagrange multiplier, and *b* is a constant.

In our work, we utilize the sequential minimal optimization (SMO) algorithm [21], which gives an efficient way of solving the dual problem of the support vector machine optimization problem.

#### 3.2.3. Multilayer Perceptron Networks

The multilayer perceptron network (MLP) [22] is a feed-forward neural network with one or more layers that are hidden from the input and output nodes. Neural networks have the ability to learn complex data structures and approximate any continuous mapping [23]. The model of each neuron in the network includes a nonlinear activation function that is differentiable such as the sigmoid. The units each perform a biased weighted sum of their inputs and pass this activation level through the transfer function to produce their output given by

(14) $$\varphi \left(x\right)=f\left(w^{T}x+\theta \right),$$

where  *w* is the synaptic vector, *x* is the input vector, *θ* is the bias constant, and *T* is the transpose operator. For *K*-class classification, the MLP uses back propagation to implement nonlinear discriminants. There are *K* outputs with softmax as the output nonlinearity.

### 3.3. Dataset Labeling and Preprocessing

Our proposed system relies on a labeled dataset, to effectively build an automated tongue-based ZHENG classification system. Our dataset is comprised of tongue images from 263 gastritis patients and a control group of 48 healthy volunteers. Most of the gastritis patients have been classified as Hot or Cold ZHENG and are identified with a color label (yellow or white) based on the color of the coating of their tongue, as determined by their Chinese doctors. The doctors also carry out a detailed profile of the ZHENG symptoms for each patient based on clinical evaluations. The list of the main symptom profile terms is summarized in Table 1. 

We are also interested in the relationship between TCM diagnosis and Western medicine diagnosis; hence, for a subset of the patients, we are provided with their corresponding Western medical gastritis pathology. They are grouped into two categories: superficial versus atrophic. In Western medicine, the doctors are also interested in knowing whether the *Helicobacter Pylori* (HP) bacterium found in the stomach is present (positive) or absent (negative) in the patients with chronic gastritis. Thus, we are provided with that information for a subset of the patients. It was not feasibleto obtain all the different information collected per patient.  Table 2 summaries the population of each subset for four different labels (ZHENG, Coating, Pathology, and HP).

## 4. Results and Analysis

### 4.1. Experimental Setup

In this section, we evaluated the performance of our proposed ZHENG classification system using the three classification models (AdaBoost, SVM, and MLP) described in Section 3.2. We compared the performance of training the classifier models using the set of features extracted from the entire tongue image versus the middle tongue region only. As mentioned in Section 2, in TCM, it is believed that the middle tongue region provides discriminant information for diagnosing stomach disorders. *Hence, we extract features from the middle tongue region, as described in Figure 1(b), to evaluate the performance compared to extracting features from the entire tongue region.* In training and testing our classification models, we employ a 3-fold cross-validation strategy. This implies that the data is split into three sets; one set is used for testing and the remaining two sets are used for training. The experiment is repeated with each of the three sets used for testing. The average accuracy of the tests over the three sets is taken as the performance measure. For each classification model, we varied the parameters to optimize its performance. We also compare the results obtained using the five different variations of the feature vector (mean = $\mu \vec{F}$, median $=\text{med}\;\vec{F}$, standard deviation = $\sigma \vec{F}$, mean + standard deviation = $\left\{\mu \vec{F},\;\sigma \vec{F}\right\}$, and median + standard deviation = $\left\{\text{med}\;\vec{F},\;\sigma \vec{F}\right\}$), as described in Section 3.1. We also apply Information Gain attribute evaluation on the feature vectors to quantify and rank the significance of individual features. *Lastly, we apply the Best First feature selection algorithm to select the “significant” features before training the classifiers to compare the performance of training the classifiers with the whole feature set against selected features.*

The performance metrics used are the classification accuracy (CA) and the average *F*-measure. CA is defined as the percentage of correctly classified instances over the entire set of instances classified. In our dataset, as described in Table 2, for each data label, the population of both classes (which we denote by {*C*_1_, *C*_2_}) is not uniformly distributed. Hence, evaluating the performance of our classifiers using simply the classification accuracy does not paint an accurate picture of the discriminative power of the classifier. Since the dataset distribution is skewed, we can achieve a high accuracy but very poor performance in discriminating between both classes. Thus, we judge our classifiers using the average *F*-measure obtained for both binary classes. The *F*-measure combines precision and recall. It measures how well an algorithm can predict an instance belonging to a particular class. Let TP represent true positive, which we define as the number of instances that are correctly classified as *C*_1_ for a given test set, while TN denotes true negative, the equivalent for *C*_2_ instances. Let FP represent false positive, which we define as the number of instances that are incorrectly classified as *C*_1_ for a given test set, while FN denotes false negative, the equivalent for *C*_2_ instances. Precision = TP/(TP + FP) and Recall = TP/(TP + FN). Thus, the *F*-measure is defined as

(15) $$F\text{-measure}=\;\frac{2\cdot \text{Recall}\cdot \text{Precision}}{\text{Recall}+\text{Precision}}.$$

For both binary classes {*C*_1_, *C*_2_}, let (|*C*_1_|, |*C*_2_|) denote the total number of instances belonging to class *C*_1_ and *C*_2_, respectively, then the average *F*-measure is defined as

(16) $$\begin{array}{cc} & \overset{̅}{F\text{-measure}}\\ & \;=\;\frac{\left|C_{1}\right|\cdot F\text{-measure}\;\left(C_{1}\right)+\left|C_{2}\right|\cdot F\text{-measure}\;\left(C_{2}\right)}{\left|C_{1}\right|+\left|C_{2}\right|}. \end{array}$$

In all the tables illustrating the different experimental results, we highlight the best $\overset{̅}{\;F\text{-measure}}$ obtained along with the corresponding classification accuracy of the classifier.

### 4.2. Classification Results Based on Tongue Coating and ZHENG for Gastritis Patients

The experimental results presented in this section analyze the discrimination among the gastritis patients based on their tongue coating color and ZHENG category.  Table 3 summarizes the results obtained using our proposed color-space feature vector to train the classifiers to automatically classify the color of the coating of a gastritis patient's tongue as yellow or white. We can observe from Table 3 that the combination of the median and standard deviation feature values ($\left\{\text{med}\;\vec{F},\;\sigma \vec{F}\right\})$ yields the best result for both the entire tongue region and the middle tongue region only. The results for both regions are also very comparable.

When using the entire tongue region, the top three significant features for the color coating classification, ranked by the information gain attribute, were {*σF*_9_, med *F*_12_, *σF*_2_}, which denote the standard deviation of *Q* chroma (YIQ model), the median of Cr component (YCbCr), and the standard deviation of Green Channel (RGB), respectively. For the middle tongue region only, the top three were {*σF*_9_, *σF*_20_, med *F*_4_} which denote the standard deviation of *Q* chroma (YIQ model), the standard deviation of *u* component (L*u*v*), and the median of the Hue (HSV). It is also interesting to observe that out of the top ten significant features using the entire region versus the middle tongue region, they both have six of those features in common.

The result obtained on ZHENG classification between the Hot and Cold groups is shown in Table 4. For the ZHENG classification, using the standard deviation feature values $\left(\sigma \vec{F}\right)$ performs best when dealing with the entire tongue region while the $\left\{\text{med}\;\vec{F},\sigma \vec{F}\right\}$ feature vector is the top performer for the middle tongue region only.

For ZHENG classification between Hot and Cold *syndromes* for gastritis patients, when using the entire tongue region, only one feature was considered significant by the information gain attribute: *σF*_9_, that is, which is the standard deviation of *Q* chroma (YIQ model). For the middle tongue region, the most important feature is *σF*_20_, the standard deviation of *u* component (L*u*v*). Even though the noteworthy feature in the entire tongue area and the middle tongue area is not the same, both *Q* components in YIQ color space and *u* component in L*u*v* color space show the difference from green to red in chromaticity diagram.

Table 5 summarizes the results obtained when we train different classifiers to detect the presence of the HP bacteria in a gastritis patient using the color feature vector. The classification result obtained in learning the pathology groups of the patients (superficial versus atrophic) is shown in Table 6. Both cases are not very strong, which illustrates a weak correlation between the western medicine diagnosis and the tongue information utilized by Chinese medical practitioners. No feature was identified as significant in either case.

Tables 7–10 illustrate how experimental results reflect the analysis of the classification *between* two pathology types of gastritis patients according to ZHENG category.  Table 7 summarizes the results obtained using our proposed color-space feature vector to train the classifiers to automatically classify between Superficial group and Atrophic group for patients labeled as Cold ZHENG. The results obtained on classification between superficial group and atrophic group for Hot ZHENG patients is shown in Table 8. We can observe from Table 7 that the $\sigma \vec{F}$ feature vector performed best for the entire tongue region while the $\left\{\text{med}\;\vec{F},\;\sigma \vec{F}\right\}$ feature vector yielded the best result for the middle tongue region.

Similarly, from Table 8 we can observe that for the Hot ZHENG patients, for the middle tongue region, the $\left\{\text{med}\;\vec{F},\;\sigma \vec{F}\right\}$ feature vector also performed best. However, $\left\{\mu \vec{F},\;\sigma \vec{F}\right\}$ feature vector performs best when dealing with the entire tongue region.

When using the entire tongue region, the top three significant features for the pathology classification between superficial and atrophic in Cold ZHENG, ranked by the information gain attribute, were {*σF*_9_, *σF*_6_, *σF*_1_} which denote the standard deviation of *Q* chroma (YIQ model), the standard deviation of value component (HSV), and the standard deviation of Red Channel (RGB), respectively. 

In Table 8, when using the entire tongue region, the top three significant features for the pathology classification between superficial and atrophic in Hot syndrome, ranked by the information gain attribute, were {*μF*_22_, *μF*_25_, *μF*_3_} which denote the mean of Cyan Ink (CMYK model), the mean of Black Ink (CMYK model), and the mean of Blue Channel (RGB), respectively. For the middle tongue region only, the top three were {*σF*_22_, *σF*_25_, med *F*_25_}, which denote the standard deviation of Cyan Ink (CMYK model), the standard deviation of Black Ink (CMYK model), and the median of Black Ink (CMYK model).

The next set of experimental results focus on training our classifier using our proposed color-space feature vector to discriminate Hot ZHENG from Cold ZHENG in each pathology group.  Table 9 summarizes the results obtained to train the classifiers to automatically classify between Hot and Cold ZHENG for superficial gastritis patients.  Table 10 reflects the results for gastritis patients. We can observe from Table 9 that both $\left\{\mu \vec{F},\;\sigma \vec{F}\right\}$ and $\left\{\text{med}\;\vec{F},\;\sigma \vec{F}\right\}$ feature vectors perform the best for both the entire tongue region and the middle tongue region. From results in Table 10, using the standard deviation feature values ($\left\{\mu \vec{F},\;\sigma \vec{F}\right\}$) performs best when dealing with the entire tongue region while the ($\left\{\mu \vec{F},\;\sigma \vec{F}\right\}$) feature vector is the top performer for the middle tongue region.

When using the entire tongue region, the top three significant features for the ZHENG classification between Hot syndrome and Cold syndrome in the patients who are superficial, ranked by the information gain attribute, were {*σF*_9_, med *F*_3_, med *F*_18_}, which denote the standard deviation of *Q* chroma (YIQ model), the median of Blue Channel (RGB), and the median of the blue sensitivity *Z* component, respectively. For the middle tongue region only, the top three were med *F*_24_, *σF*_19_, and med *F*_5_ which denote the median of Yellow Ink (CMYK), the standard deviation of lightness component (Luv model), and the median of saturation (HSV). It is also interesting to observe that by comparing the set of the top five significant features using the entire region versus the set from the middle tongue region, they both have the Yellow Ink (CMYK) in common.

When using the entire tongue region, there is only one significant feature difference for the ZHENG classification between *Hot syndrome and Cold syndrome* in patients who are atrophic, ranked by the information gain attribute, *σF*_9_ which denotes the standard deviation of *Q* chroma (YIQ model). For the middle tongue region only, there were two significant features: {*μF*_19_, *μF*_3_} which denote the mean of the blue sensitivity *Z* component (XYZ) and the mean of the Blue Channel (RGB). 

### 4.3. Classification Results for Gastritis Patients versus Control Group

The experimental results presented in this section analyze the discrimination between the gastritis patients and control group.  Table 11 summarizes the results obtained using our proposed color-space feature vector to train the classifiers to automatically classify patients with coating on tongue versus healthy patients with normal tongue (without coating). We can observe from Table 11 that the $\left\{\text{med}\;\vec{F},\;\sigma \vec{F}\right\}$ feature vector yields the best result for the entire tongue region while for the middle tongue region, it was the $\sigma \vec{F}$ feature vector. 

When using the entire tongue region, the top three significant features for distinguishing between normal tongue and tongue with coating, ranked by the information gain attribute, were {*σF*_1_, *σF*_6_, *σF*_25_} which denote the standard deviation of Red Channel (RGB), the standard deviation of value component (HSV), and the standard deviation of Black Ink (CMYK) respectively. For the middle tongue region only, there were only two significant features: {*σF*_13_, *σF*_14_} which denote the standard deviation of lightness component (L*a*b) and the standard deviation of a* component (L*a*b*). It is also interesting to observe that by comparing the set of the top 10 significant features using the entire region versus the set from the middle tongue region, they both have the lightness and a* component (L*a*b*) in common.

The results obtained from the classification between the normal group and the entire set of patients with ZHENG syndrome is shown in Table 12. The $\left\{\mu \vec{F},\;\sigma \vec{F}\right\}$ feature vector performs best when dealing with the entire tongue region while the $\left\{\text{med}\;\vec{F},\;\sigma \vec{F}\right\}$ feature vector is the top performer for the middle tongue region.

When using the entire tongue region, the top three significant features for the classification between the normal group and the gastritis group, ranked by the information gain attribute, were {*σF*_1_, *σF*_6_, *σF*_25_} which denote the standard deviation of Red Channel (RGB), the standard deviation of value component (HSV), and the standard deviation of Black Ink (CMYK) respectively. For the middle tongue region only, the top three were: {med *F*_1_, med *F*_6_, *σF*_13_} which denote the median of Red Channel (RGB), the median of Value component (HSV), and the standard deviation of lightness component (L*a*b*). 

Tables 13 and 14 show the results of training our classifiers to discriminate between the normal group and the Hot ZHENG patients only, and then normal group versus Cold ZHENG patients only.  Table 13 illustrates the results for normal versus hot ZHENG. We can observe that the $\sigma \vec{F}$ feature vector performs best both for the entire tongue region and the middle tongue region. From Table 14, when only the normal versus Cold ZHENG patients is considered, the same feature vector, $\left\{\mu \vec{F},\;\sigma \vec{F}\right\}$, performs best for both cases, however, considering only the middle tongue region outperforms using the entire tongue region.

When using the entire tongue region, the top three significant features for the classification between the normal group and the gastritis *patients with Hot syndrome*, ranked by the information gain attribute, were {*σF*_1_, *σF*_6_, *σF*_25_} which denote the standard deviation of Red Channel (RGB), the standard deviation of value component (HSV), and the standard deviation of Black Ink (CMYK), respectively. For the middle tongue region only, there were only two significant features: {*σF*_13_, *σF*_14_} which denote the standard deviation of lightness component (L*a*b) and the standard deviation of a* component (L*a*b*). When the set of the top ten significant features using the entire region versus the set from the middle tongue region are compared, they both have the lightness and a* component (L*a*b*) in common. 

When using the entire tongue region, the top three significant features for the classification between the normal group and the gastritis *patients with Cold syndrome*, ranked by the information gain attribute, were {*σF*_25_, *σF*_22_, *σF*_1_} which denotethe standard deviation of Black Ink (CMYK), the standard deviation of Cyan Ink (CMYK), and the standard deviation of Red Channel (RGB), respectively. For the middle tongue region only, the top three were {*σF*_13_, *μF*_22_, *σF*_14_} which denote the standard deviation of lightness component (L*a*b), the mean of Cyan Ink (CMYK), and the standard deviation of a* component (L*a*b*). 

Table 15 show the results of training our classifiers to discriminate between the normal group and the superficial patients while Table 16 shows the result for normal group versus the atrophic patients. When using the entire tongue region, the top three significant features for the classification between the normal group and the superficial group, ranked by the information gain attribute, were {*σF*_1_, *σF*_6_, *σF*_25_} which denote the standard deviation of Red Channel (RGB), the standard deviation of value component (HSV), and the standard deviation of Black Ink (CMYK), respectively. For the middle tongue region, the top three were {med*F*_9_, med*F*_1_, med*F*_6_} which denote the median of *Q* chromatic component (YIQ), the median of Red Channel (RGB), and the median of Value component (HSV). 

When using the entire tongue region, the top three significant features for the classification between the normal group and the atrophic group, ranked by the information gain attribute, were {*μF*_25_, *μF*_22_, *μF*_1_} which denote the mean of Black Ink (CMYK model), the mean of Cyan Ink (CMYK model), and the mean of Red Channel (RGB), respectively. For the middle tongue region, the top three were {med *F*_16_, *σF*_13_, *σF*_23_} which denote the median of red sensitivity *X* component (XYZ), the standard deviation of lightness (L*a*b*), and the standard deviation of Cyan Ink (CMYK).

### 4.4. Analysis of Classification Results

From the experimental results presented in Sections 4.2 and 4.3, we can draw the following conclusions. Firstly, concerning the performance of the different classification models, we observe that the MLP and SVM models usually outperformed the AdaBoost model. The multilayer perceptron neural network seems most adequate for learning the complex relationships between the color features of the tongue images and the ZHENG/coating classes. However, both the MLP and SVM models have many parameters to consider and optimize while the AdaBoost is a much simpler model. In the AdaBoost model, we use a decision tree as our base weak learner and vary the number of classifiers to optimize its performance. 

Secondly, we observe that when making discriminations within the gastritis patients group (hot versus cold ZHENG, yellow versus white coating, etc.), it was more profitable to apply the feature vectors on the entire tongue image. When classifying the normal groups versus the ZHENG groupings, usually, it improved classifier performance to apply the feature vectors to the middle tongue regions only. 

Thirdly, we also observe that from the evaluation of the variations of the feature vectors used, taking into account both the average and the standard deviation usually resulted in an excellent performance. It seemed like the mean outperformed the median slightly, overall, that is, $\left\{\mu \vec{F},\;\sigma \vec{F}\right\}$. In a few cases, simply considering variation of the spread of the values over the region ($\left\{\sigma \vec{F}\right\}$) yielded the best performance. Thus, we can conclude that when deriving a feature vector for the tongue image, the mean (or median) as well as the standard deviation (which takes into account the variation of the spread on the region) is very important.

Lastly, we observe that though we were not able to effectively discriminate between the pathology groups (superficial versus atrophic and also the presence of the HP bacterium using our color-space feature vectors, we were able to classify them much better when we took into account the ZHENG classes. This further strengthens the notion that our proposed color-space feature vectors are able to discriminate between the hot and cold ZHENG patients in addition to discerning a ZHENG patient from a non-ZHENG (healthy) patient.

### 4.5. Applying Feature Selection Algorithm

The classification results presented in Sections 4.2 and 4.3 were obtained using the whole feature set. For each experiment carried out on the entire tongue region, we also applied information gain attribute evaluation to rank the significance of the features. In this section, we apply feature selection algorithm (Best First) to select only a subset of features, which are deemed significant, before training the classifiers. Our goal is to see if this would yield a better result than using the whole feature set. The Best First algorithm searches the space of attribute subsets by greedy hill climbing augmented with a backtracking facility.

The summary of the results obtained is shown in Table 17. The normal group refers to the healthy (non-ZHENG) control group. We present the best classification result obtained for each experiment based on using the five variations of the feature vectors ($\mu \vec{F}$, $\text{med}\;\vec{F}$, $\sigma \vec{F}$, $\left\{\mu \vec{F},\;\sigma \vec{F}\right\}$, $\left\{\text{med}\;\vec{F},\;\sigma \vec{F}\right\}$) and the three different classification models (Adaboost, SVM, and MLP). As we can observe from Table 17, using the whole feature set to train the classifiers yielded a better result in all cases except for the Atrophic Patients (Hot versus Cold ZHENG) experiment. Thus, we can conclude the overall, using the aggregate of the proposed feature sets is more discriminative even though some features are more significant than others.

## 5. Conclusion and Future Work

In this paper, we propose a novel color space-based feature set for use in the clinical characterization of ZHENG using various supervised machine-learning algorithms. Using an automated tongue-image diagnosis system, we extract these objective features from tongue images of clinical patients and analyze the relationship with their corresponding ZHENG data and disease prognosis (specifically gastritis) obtained from clinical practitioners. Given that TCM practitioners usually observe the tongue color and coating to determine ZHENG (such as Cold or Hot ZHENG) and to diagnose different stomach disorders including gastritis. We propose using machine-learning techniques to establish the relationship between the tongue image features and ZHENG by learning through examples. 

The experimental results obtained demonstrate an excellent performance of our proposed system. *Our future work will focus on improving the performance of our system by exploring additional tongue image features that can be extracted to further strengthen our classification models. We plan to explore ways to improve our methodology to more accurately classify the ZHENGs such as including a preprocessing step of coating separation prior to the feature extraction phase. Lastly, we plan to evaluate the classification of the other ZHENG types mentioned in Section 1.*

## Figures and Tables

**Table 1:** Symptom profile terms of Cold ZHENG and Hot ZHENG.

Subjects	Terms (keywords)
Cold-ZHENG related symptoms	Cold (chill, coldness), hot diet/drink preferred, desires warm environment, pale flushing of face, not thirsty, no bad mouth breath, no acidic saliva, clear urine, loose stool, high and short pitch voice, and feeling cold at limbs.
Hot-ZHENGrelated symptoms	Fever (heat, hot), cold diet/drink preferred, desires cold environment, red flushing of face, thirsty, obvious bad mouth breath, acidic saliva, yellow urine, hard stool, constipation, and feeling hot at limbs.

**Table 2:** Data label summary for the gastritis patients.

Data labels	Population
ZHENG: Hot/Cold	132/68
Coating: yellow/white	147/67
Pathology: superficial/atrophic	84/144
HP bacterium: positive/negative	72/167

**Table 3:** Tongue coating color classification: yellow versus white for gastritis patients.

Feature vector	Entire tongue						Middle tongue					
	AdaBoost		SVM		MLP		AdaBoost		SVM		MLP	
	*F*-meas	CA	*F*-meas	CA	*F*-meas	CA	*F*-meas	CA	*F*-meas	CA	*F*-meas	CA
$\mu \vec{F}$	0.681	69.16	0.757	76.64	0.752	76.17	0.761	77.57	0.796	80.84	0.773	78.04
$\left\{\mu \vec{F},\;\sigma \vec{F}\right\}$	0.743	74.77	0.792	79.44	0.774	77.57	0.764	76.64	0.799	80.37	0.767	77.10
$\text{med}\;\vec{F}$	0.758	76.64	0.728	74.30	0.724	72.90	0.735	74.77	0.789	79.44	0.766	77.10
$\left\{\text{med}\;\vec{F},\;\sigma \vec{F}\right\}$	0.763	76.64	**0.801**	**80.37**	0.767	77.10	0.781	78.50	0.775	77.10	**0.811**	**81.31**
$\sigma \vec{F}$	0.747	75.70	0.797	79.91	0.783	78.50	0.747	74.77	0.777	77.57	0.783	78.97

**Table 4:** ZHENG classification between Hot and Cold syndromes for gastritis patients.

Feature vector	Entire tongue						Middle tongue					
	AdaBoost		SVM		MLP		AdaBoost		SVM		MLP	
	*F*-meas	CA	*F*-meas	CA	*F*-meas	CA	*F*-meas	CA	*F*-meas	CA	*F*-meas	CA
$\mu \vec{F}$	0.618	63.50	0.716	71.50	0.710	71.00	0.622	63.50	0.710	70.50	0.663	67.00
$\left\{\mu \vec{F},\;\sigma \vec{F}\right\}$	0.750	75.00	0.680	67.50	0.723	72.00	0.664	68.00	0.735	73.50	0.740	74.00
$\text{med}\;\vec{F}$	0.647	65.50	0.649	64.50	0.676	68.00	0.684	71.00	0.661	67.00	0.690	69.00
$\left\{\text{med}\;\vec{F},\;\sigma \vec{F}\right\}$	0.738	74.50	0.665	66.00	0.726	72.50	0.685	70.00	0.708	72.00	**0.761**	**76.00**
$\sigma \vec{F}$	**0.763**	**76.50**	0.709	71.00	0.709	71.00	0.676	69.00	0.704	70.00	0.719	72.00

**Table 5:** Detection of presence of HP bacteria (positive versus negative) in gastritis patients.

Feature vector	Entire tongue						Middle tongue					
	AdaBoost		SVM		MLP		AdaBoost		SVM		MLP	
	*F*-meas	CA	*F*-meas	CA	*F*-meas	CA	*F*-meas	CA	*F*-meas	CA	*F*-meas	CA
$\mu \vec{F}$	0.679	71.97	0.681	68.20	0.673	68.20	0.696	71.97	0.686	70.29	0.682	70.29
$\left\{\mu \vec{F},\;\sigma \vec{F}\right\}$	0.644	66.11	0.680	67.78	**0.713**	**71.97**	0.632	64.85	0.681	68.20	0.681	67.78
$\text{med}\;\vec{F}$	0.655	67.78	0.666	67.36	0.666	67.78	**0.699**	**71.55**	0.644	69.04	0.676	68.20
$\left\{\text{med}\;\vec{F},\;\sigma \vec{F}\right\}$	0.655	67.78	0.686	68.20	0.695	69.87	0.633	65.27	0.631	64.44	0.684	68.20
$\sigma \vec{F}$	0.661	68.20	0.695	71.13	0.702	70.29	0.594	61.09	0.669	66.95	0.649	65.27

**Table 6:** Classification between superficial and atrophic pathology of the gastritis patients.

Feature vector	Entire tongue						Middle tongue					
	AdaBoost		SVM		MLP		AdaBoost		SVM		MLP	
	*F*-meas	CA	*F*-meas	CA	*F*-meas	CA	*F*-meas	CA	*F*-meas	CA	*F*-meas	CA
$\mu \vec{F}$	0.604	63.16	0.642	64.47	0.627	63.16	**0.658**	**66.67**	0.631	63.16	0.622	62.72
$\left\{\mu \vec{F},\;\sigma \vec{F}\right\}$	0.633	65.35	0.662	65.79	**0.702**	**71.05**	0.604	61.40	0.630	63.60	0.621	62.28
$\text{med}\;\vec{F}$	0.633	64.47	0.601	62.72	0.640	64.04	0.623	65.79	0.632	63.16	0.623	62.28
$\left\{\text{med}\;\vec{F},\;\sigma \vec{F}\right\}$	0.657	66.23	0.660	65.79	0.697	69.74	0.613	62.72	0.645	64.47	0.663	66.23
$\sigma \vec{F}$	0.637	64.91	0.697	70.18	0.659	66.23	0.631	64.04	0.629	63.16	0.639	64.47

**Table 7:** Tongue Classification between superficial and atrophic in Cold syndrome patients.

Feature vector	Entire tongue						Middle tongue					
	AdaBoost		SVM		MLP		AdaBoost		SVM		MLP	
	*F*-meas	CA	*F*-meas	CA	*F*-meas	CA	*F*-meas	CA	*F*-meas	CA	*F*-meas	CA
$\mu \vec{F}$	0.579	58.33	0.658	66.67	0.633	63.33	0.651	65.00	0.639	65.00	0.633	63.33
$\left\{\mu \vec{F},\;\sigma \vec{F}\right\}$	0.716	71.67	0.647	65.00	0.680	68.33	0.643	65.00	0.649	65.00	0.662	66.67
$\text{med}\;\vec{F}$	0.600	60.00	0.714	71.67	0.733	73.33	0.633	63.33	0.613	66.67	0.633	63.33
$\left\{\text{med}\;\vec{F},\;\sigma \vec{F}\right\}$	0.717	71.67	0.698	70.00	0.700	70.00	**0.684**	**68.33**	0.598	60.00	0.667	66.67
$\sigma \vec{F}$	0.701	70.00	**0.761**	**76.67**	0.745	75.00	0.579	58.33	0.598	60.00	0.601	60.00

**Table 8:** Tongue classification between superficial and atrophic in Hot syndrome patients.

Feature vector	Entire tongue						Middle tongue					
	AdaBoost		SVM		MLP		AdaBoost		SVM		MLP	
	*F*-meas	CA	*F*-meas	CA	*F*-meas	CA	*F*-meas	CA	*F*-meas	CA	*F*-meas	CA
$\mu \vec{F}$	0.768	77.06	0.755	75.23	0.735	73.39	0.710	71.56	0.735	76.15	0.680	67.89
$\left\{\mu \vec{F},\;\sigma \vec{F}\right\}$	0.741	74.31	**0.845**	**84.40**	0.764	76.15	0.680	68.81	0.777	77.06	0.780	77.98
$\text{med}\;\vec{F}$	0.718	72.48	0.708	72.48	0.718	71.56	0.686	68.81	0.706	70.64	0.736	73.39
$\left\{\text{med}\;\vec{F},\;\sigma \vec{F}\right\}$	0.715	71.56	0.817	81.65	0.815	81.65	0.672	67.89	0.774	77.06	**0.808**	**80.73**
$\sigma \vec{F}$	0.770	77.06	0.818	81.65	0.817	81.65	0.675	67.89	0.792	78.90	0.781	77.98

**Table 9:** Tongue classification between Hot syndrome and Cold syndrome in superficial patients.

Feature vector	Entire tongue						Middle tongue					
	AdaBoost		SVM		MLP		AdaBoost		SVM		MLP	
	*F*-meas	CA	*F*-meas	CA	*F*-meas	CA	*F*-meas	CA	*F*-meas	CA	*F*-meas	CA
$\mu \vec{F}$	0.583	59.68	0.773	77.42	0.705	70.97	0.705	70.97	0.773	77.42	0.726	72.58
$\left\{\mu \vec{F},\;\sigma \vec{F}\right\}$	0.740	74.19	**0.839**	**83.87**	0.765	77.42	0.690	69.35	**0.839**	**83.87**	0.757	75.81
$\text{med}\;\vec{F}$	0.628	62.90	0.740	74.19	0.743	74.19	0.675	67.74	0.710	70.97	0.658	66.13
$\left\{\text{med}\;\vec{F},\;\sigma \vec{F}\right\}$	0.774	77.42	**0.839**	**83.87**	0.755	75.81	0.774	77.42	**0.839**	**83.87**	0.774	77.42
$\sigma \vec{F}$	0.834	83.87	0.757	75.81	0.838	83.87	0.819	82.26	0.791	79.03	0.750	75.81

**Table 10:** Tongue Classification between Hot syndrome and Cold syndrome in atrophic patients.

Feature vector	Entire tongue						Middle tongue					
	AdaBoost		SVM		MLP		AdaBoost		SVM		MLP	
	*F*-meas	CA	*F*-meas	CA	*F*-meas	CA	*F*-meas	CA	*F*-meas	CA	*F*-meas	CA
$\mu \vec{F}$	0.539	55.14	0.642	63.55	0.645	64.49	0.572	58.88	**0.762**	**75.70**	0.615	61.68
$\left\{\mu \vec{F},\;\sigma \vec{F}\right\}$	0.662	67.29	0.681	69.16	0.698	70.09	0.638	64.49	0.702	69.16	0.685	68.22
$\text{med}\;\vec{F}$	0.612	61.68	0.646	63.55	0.666	66.36	0.611	62.62	0.606	62.62	0.638	64.49
$\left\{\text{med}\;\vec{F},\;\sigma \vec{F}\right\}$	0.704	71.03	0.657	64.49	0.677	68.22	0.604	60.75	0.701	69.16	0.703	70.09
$\sigma \vec{F}$	0.696	70.09	0.691	68.22	**0.734**	**73.83**	0.650	64.49	0.675	66.36	0.645	63.55

**Table 11:** Classification between normal tongue and tongue with coating.

Feature vector	Entire tongue						Middle tongue					
	AdaBoost		SVM		MLP		AdaBoost		SVM		MLP	
	*F*-meas	CA	*F*-meas	CA	*F*-meas	CA	*F*-meas	CA	*F*-meas	CA	*F*-meas	CA
$\mu \vec{F}$	0.803	82.82	0.831	82.44	0.795	80.53	0.771	78.63	0.774	77.48	0.764	75.95
$\left\{\mu \vec{F},\;\sigma \vec{F}\right\}$	0.829	83.59	0.851	85.11	0.848	85.50	0.812	81.68	0.814	81.68	0.816	82.44
$\text{med}\;\vec{F}$	0.785	80.53	0.803	83.21	0.814	83.21	0.776	80.53	0.791	78.63	0.784	79.39
$\left\{\text{med}\;\vec{F},\;\sigma \vec{F}\right\}$	0.814	83.21	0.835	83.59	**0.861**	**86.26**	0.817	83.59	0.823	82.06	0.824	82.44
$\sigma \vec{F}$	0.818	83.21	0.839	83.59	0.851	85.11	**0.837**	**84.73**	0.786	79.39	0.818	82.44

**Table 12:** Tongue classification between normal group and ZHENG gastritis group.

Feature vector	Entire tongue						Middle tongue					
	AdaBoost		SVM		MLP		AdaBoost		SVM		MLP	
	*F*-meas	CA	*F*-meas	CA	*F*-meas	CA	*F*-meas	CA	*F*-meas	CA	*F*-meas	CA
$\mu \vec{F}$	0.765	78.63	0.809	80.24	0.784	78.63	0.781	79.44	0.770	76.61	0.762	76.61
$\left\{\mu \vec{F},\;\sigma \vec{F}\right\}$	0.836	84.68	0.852	84.68	**0.857**	**85.89**	0.820	82.66	0.798	80.65	0.826	82.26
$\text{med}\;\vec{F}$	0.756	77.82	0.795	81.45	0.784	78.63	0.772	78.23	0.817	81.45	0.785	78.63
$\left\{\text{med}\;\vec{F},\;\sigma \vec{F}\right\}$	0.802	81.45	0.845	84.27	0.844	84.68	0.779	79.44	0.837	83.47	**0.869**	**87.10**
$\sigma \vec{F}$	0.826	83.47	0.849	84.68	0.843	84.27	0.799	81.05	0.780	77.02	0.833	83.87

**Table 13:** Tongue classification between normal group and Hot ZHENG.

Feature vector	Entire tongue						Middle tongue					
	AdaBoost		SVM		MLP		AdaBoost		SVM		MLP	
	*F*-meas	CA	*F*-meas	CA	*F*-meas	CA	*F*-meas	CA	*F*-meas	CA	*F*-meas	CA
$\mu \vec{F}$	0.671	70.00	0.781	77.78	0.708	72.22	0.741	75.00	0.773	77.22	0.755	76.11
$\left\{\mu \vec{F},\;\sigma \vec{F}\right\}$	0.804	80.56	0.792	79.44	0.816	81.67	0.780	78.89	0.764	77.22	0.799	79.44
$\text{med}\;\vec{F}$	0.721	72.78	0.711	72.22	0.739	75.00	0.727	73.89	0.739	73.33	0.744	74.44
$\left\{\text{med}\;\vec{F},\;\sigma \vec{F}\right\}$	0.796	80.00	0.814	82.78	0.797	80.00	0.781	79.44	0.752	75.00	0.798	79.44
$\sigma \vec{F}$	0.768	77.22	**0.828**	**82.22**	0.826	82.78	0.736	75.00	0.766	77.22	**0.805**	**80.56**

**Table 14:** Tongue classification between normal group and Cold ZHENG.

Feature vector	Entire tongue						Middle tongue					
	AdaBoost		SVM		MLP		AdaBoost		SVM		MLP	
	*F*-meas	CA	*F*-meas	CA	*F*-meas	CA	*F*-meas	CA	*F*-meas	CA	*F*-meas	CA
$\mu \vec{F}$	0.690	68.97	0.759	75.86	0.676	68.10	0.714	71.55	0.741	74.14	0.731	73.28
$\left\{\mu \vec{F},\;\sigma \vec{F}\right\}$	0.742	74.14	**0.785**	**78.45**	0.748	75.00	**0.826**	**82.76**	0.759	75.86	0.750	75.00
$\text{med}\;\vec{F}$	0.686	68.97	0.745	75.00	0.757	75.86	0.672	67.24	0.750	75.00	0.742	74.14
$\left\{\text{med}\;\vec{F},\;\sigma \vec{F}\right\}$	0.759	75.86	0.774	77.59	0.734	73.28	0.768	76.72	0.733	73.28	0.811	81.03
$\sigma \vec{F}$	0.741	74.14	0.733	73.28	0.734	73.28	0.679	68.10	0.723	72.41	0.708	70.69

**Table 15:** Tongue classification between normal group and superficial patients.

Feature vector	Entire tongue						Middle tongue					
	AdaBoost		SVM		MLP		AdaBoost		SVM		MLP	
	*F*-meas	CA	*F*-meas	CA	*F*-meas	CA	*F*-meas	CA	*F*-meas	CA	*F*-meas	CA
$\mu \vec{F}$	0.655	65.91	0.737	74.24	0.754	75.76	0.694	69.70	0.687	68.18	0.704	70.45
$\left\{\mu \vec{F},\;\sigma \vec{F}\right\}$	0.679	68.18	0.751	75.00	0.774	77.27	0.749	75.00	0.744	74.24	0.719	71.97
$\text{med}\;\vec{F}$	0.675	67.42	0.737	74.24	0.737	73.48	0.733	73.48	0.677	67.42	0.739	73.48
$\left\{\text{med}\;\vec{F},\;\sigma \vec{F}\right\}$	0.695	70.45	0.759	75.76	**0.811**	**81.06**	0.749	75.00	**0.762**	**75.76**	0.726	72.73
$\sigma \vec{F}$	0.687	68.94	0.735	74.24	0.706	70.45	0.726	72.73	0.742	74.24	0.749	75.00

**Table 16:** Tongue classification between normal group and atrophic patients.

Feature vector	Entire tongue						Middle tongue					
	AdaBoost		SVM		MLP		AdaBoost		SVM		MLP	
	*F*-meas	CA	*F*-meas	CA	*F*-meas	CA	*F*-meas	CA	*F*-meas	CA	*F*-meas	CA
$\mu \vec{F}$	0.733	75.52	0.803	80.21	0.781	79.17	0.754	77.08	0.770	78.13	0.699	70.83
$\left\{\mu \vec{F},\;\sigma \vec{F}\right\}$	0.736	73.96	0.772	78.13	**0.837**	**83.85**	0.798	80.73	0.782	78.65	0.802	80.21
$\text{med}\;\vec{F}$	0.726	73.96	0.754	77.08	0.751	75.52	0.726	75.52	0.749	74.48	0.753	75.52
$\left\{\text{med}\;\vec{F},\;\sigma \vec{F}\right\}$	0.738	74.48	0.816	82.29	0.818	81.77	0.751	75.52	0.792	78.65	**0.848**	**84.90**
$\sigma \vec{F}$	0.761	77.08	0.787	79.69	0.799	80.21	0.772	78.13	0.798	80.21	0.791	79.69

**Table 17:** Comparison between using selected features versus Whole feature set for classification.

Classification experiment type	Feature selection		Whole feature	
	*F*-measure	Accuracy	*F*-measure	Accuracy
Coating (yellow versus white)	0.764	77.10%	0.801	80.37%
ZHENG (Hot versus Cold)	0.642	65.00%	0.763	76.50%
HP Bacteria (positive versus negative)	0.636	72.38%	0.713	71.97%
Gastritis patients (superficial versus atrophic)	0.656	68.42%	0.702	71.05%
Cold ZHENG patients (superficial versus atrophic)	0.750	75.00%	0.761	76.67%
Hot ZHENG patients (superficial versus atrophic)	0.776	77.98%	0.845	84.40%
Superficial Patients (Hot versus Cold ZHENG)	0.807	80.65%	0.839	83.87%
Atrophic patients (Hot versus Cold ZHENG)	**0.782**	**78.50%**	**0.734**	**73.83%**
Normal tongue versus tongue with coating	0.833	85.88%	0.861	86.26%
Normal group versus ZHENG patients	0.834	84.68%	0.857	85.89%
Normal group versus Hot ZHENG	0.808	81.11%	0.828	82.22%
Normal group versus Cold ZHENG	0.750	75.00%	0.785	78.45%
Normal group versus superficial patients	0.765	76.52%	0.811	81.06%
Normal group versus atrophic patients	0.762	78.13%	0.837	83.85%
